# Polymorphonuclear cell surface expression patterns differ in inflammatory and infectious stages in polytraumatized and septic shock patients

**DOI:** 10.1186/cc13418

**Published:** 2014-03-17

**Authors:** M Weiss, Z Gueldue, M Georgieff, F Gebhard, M Huber-Lang, M Schneider

**Affiliations:** 1 University Hospital Medical School, Ulm, Germany

## Introduction

Each cell of the immune system shows a characteristic expression of cell surface molecules depending on the grade of activation or suppression. We examined the cell surface profile on polymorphonuclear cells (PMN) of polytraumatized patients and compared them with those of healthy controls and furthermore with patients with severe sepsis or septic shock. Moreover, we wanted to find out in the polytraumatized patients whether there are associations of PMN surface marker patterns with severity of injury, of disease and of organ dysfunctions.

## Methods

In a prospective observational study, cell surface expression of CD16, CD88, CD64, CD66b, CD11b, CD95, CD33, CD39, IL-17RA, TLR- 2, TLR-4 and HLA-DR on PMN was determined using flow cytometry. Thirteen healthy volunteers, eight patients with severe sepsis and septic shock, and longitudinally, on admission (0 hours), and 4, 12, 24, 48, 120 and 240 hours thereafter, eight polytraumatized patients were monitored. ISS, SAPS3 and SOFA score reflect severity of injury, of disease and of organ dysfunctions.

## Results

In polytraumatized patients (24 hours measurement), but also in septic patients, CD88^+ ^expression and CD33^+^/CD66b^− ^expression were significantly lower and CD33/CD66b^+ ^higher than in healthy controls. Especially, the lowest values of CD88^+ ^occurred in patients with polytrauma developing septic complications.

Furthermore, the polytraumatized patients revealed higher levels of CD66b, CD11b and CD16, whereas CD16^+ ^was lower. Considering the post-traumatic course, polytraumatized patients with better prognostic evaluation in SOFA andSAPS3 initially expressed higher levels of TLR-2. Polytrauma patients could be divided into two subgroups, one with and the other without septic complications. The septic subgroup presented predominantly higher values in SOFA, SAPS3 and ISS scoring.

## Conclusion

Cell surface molecules on PMN of polytraumatized patients differ from those of healthy volunteers. Besides, distinct expression patterns resemble those of patients with sepsis. Patents with higher ISS values go along with higher scores in SOFA and SAPS3, and are at risk to develop septic complications.

